# Contribution of FEF to Attentional Periodicity during Visual Search: A TMS Study

**DOI:** 10.1523/ENEURO.0357-18.2019

**Published:** 2019-06-24

**Authors:** Laura Dugué, Alexy-Assaf Beck, Philippe Marque, Rufin VanRullen

**Affiliations:** 1 Centre National de la Recherche Scientifique (CNRS), Unité Mixte de Recherche 8002, 75006 Paris, France; 2 Université Paris Descartes, Sorbonne Paris Cité, Integrative Neuroscience and Cognition Center, 75006 Paris, France; 3 Psychological Sciences Research Institute and Institute of Neuroscience, Université de Louvain, B-1348 Louvain-la-Neuve, Belgique; 4 Médecine Physique et de Réadaption, 31059 Toulouse, France; 5 CNRS, Unité Mixte de Recherche 5549, Faculté de Médecine de Purpan, 31052 Toulouse, France; 6 Université de Toulouse, Centre de Recherche Cerveau et Cognition, Université Paul Sabatier, 31052 Toulouse, France

**Keywords:** FEF, periodicity, theta, TMS, V1, visual search

## Abstract

Visual search, looking for a target embedded among distractors, has long been used to study attention. Current theories postulate a two-stage process in which early visual areas perform feature extraction, whereas higher-order regions perform attentional selection. Such a model implies iterative communication between low- and high-level regions to sequentially select candidate targets in the array, focus attention on these elements, and eventually permit target recognition. This leads to two independent predictions: (1) high-level, attentional regions and (2) early visual regions should both be involved periodically during the search. Here, we used transcranial magnetic stimulation (TMS) applied over the frontal eye field (FEF) in humans, known to be involved in attentional selection, at various delays while observers performed a difficult, attentional search task. We observed a periodic pattern of interference at ∼6 Hz (theta) suggesting that the FEF is periodically involved during this difficult search task. We further compared this result with two previous studies (Dugué et al., 2011, 2015a) in which a similar TMS procedure was applied over the early visual cortex (V1) while observers performed the same task. This analysis revealed the same pattern of interference, i.e., V1 is periodically involved during this difficult search task, at the theta frequency. Past V1 evidence reappraised for this paper, together with our current FEF results, confirm both of our independent predictions, and suggest that difficult search is supported by low- and high-level regions, each involved periodically at the theta frequency.

## Significance Statement

Attention models postulate a two-stage process during visual search in which early visual regions perform feature extraction, while higher-order regions perform attentional selection, these two levels iteratively (periodically) communicating until target recognition. Using TMS, we tested whether there is a causal link between these brain regions and attentional search performance. Similar to past V1 evidence reappraised in this study, we showed that difficult attention search is supported in the FEF by periodic processing at the theta frequency (∼6 Hz). Together, these two findings support the idea that difficult search tasks are processed by a hierarchical system involving low- and high-level regions, each involved periodically, and allowing successful attentional exploration. Nonetheless, their potential interactions remain to be demonstrated.

## Introduction

Covert attention selectively enhances visual processing at the attended location in the absence of eye movement. In the past decade, researchers studying the temporal dynamics of visual information processing have proposed that attention samples visual information periodically at low frequencies, theta (5–7 Hz; VanRullen et al., 2007; Busch and VanRullen, 2010; Landau and Fries, 2012; Fiebelkorn et al., 2013, 2018; VanRullen, 2013; Song et al., 2014; Huang et al., 2015; Landau et al., 2015; Dugué et al., 2015a,b, 2016, 2017; Helfrich et al., 2018) and alpha (8–12 Hz; Dugué and VanRullen, 2014; van Diepen et al., 2016; for review, see VanRullen, 2016). Critically, it has been proposed that the distinction between theta and alpha periodicity comes from the spatial exploration of the visual scene by attention (Dugué and VanRullen, 2017). In other words, when attention is not critical for the task, visual information is processed at the alpha frequency, whereas when attention explores the visual space (e.g., in cueing or visual search tasks), then visual information is processed at the theta frequency.

Visual search tasks, in which observers look for a target embedded among distractors, have long been used to study attentional deployment (for review, see: Eckstein, 2011; Nakayama and Martini, 2011). In search tasks known as difficult, authors have proposed a hierarchical processing stream (Palmer et al., 1993; Treisman, 1998; Itti and Koch, 2001; Deco et al., 2002). An early stage, presumably supported by early visual areas, would decompose the visual scene in given features (e.g., color, orientation, etc.). A high-level stage would then perform attentional selection, i.e., a priority map would select the spatial location of a candidate target to focus attentional resources on. The facilitation of target processing would then occur by sending feedback connections (Juan and Walsh, 2003; Saalmann et al., 2007; Dugué et al., 2011, 2015a) to the corresponding retinotopic region (Motter, 1994; Mehta et al., 2000; Schroeder et al., 2001; Kastner and Pinsk, 2004; Bressler et al., 2008). In such a model, this selection would iterate until target recognition, making two independent predictions: (1) high-level, attentional regions and (2) early visual regions both entail periodic processing during difficult, attentional search (Dugué et al., 2015a; Dugué and VanRullen, 2017). We here directly tested the first of these two predictions for the right frontal eye field (FEF), and reappraised previously published data from visual cortex (V1) to emphasize and recontextualize in a new framework the second prediction.

We used transcranial magnetic stimulation (TMS) applied over the FEF, known to be involved in attentional selection (Kastner et al., 1999; Corbetta and Shulman, 2002), at various delays while observers performed a difficult, attentional search task. We compared the results to two previously published studies (Dugué et al., 2011, 2015a) using the same difficult search task (finding the letter T among L letters) while observers were stimulated over the occipital pole (V1/V2) using a similar TMS protocol (for a comprehensive review, see Table 1). We found that, as observed for previous V1 results, the FEF is periodically involved during the difficult search task, at the theta frequency (∼6 Hz).

**Table 1.** TMS studies investigating the role of attention during difficult, visual search tasks

		**Stimulation parameters**					
**Study**	**Behavioral manipulation**	**Test region**	**Control**	**Type of stimulation**	**Stimulation intensity**	***n***	**Conclusions**
Ashbridge (1997)	Feature: color Conjunction: color-orientation	R-PC	No-TMS	Single-pulse at 11 possible delays (from 0 to 200 ms)	80% MSI	5	R-PC involved in conjunction but not feature tasks
Walsh et al. (1998)	Conjunction: color-orientation	R-PC	No-TMS	Single-pulse at 11 possible delays (from 0 to 200 ms)	80% MSI	3	R-PC involved in novel but not learned conjunction tasks
Juan and Walsh (2003)	Feature: color Conjunction: color-orientation	V1/V2	No-TMS	10 Hz for 500 ms and Double-pulse (40 or 100 ms interval) at 6 possible delays	65% MSI	8, 6, & 6	V1 involved at late delays (feedback) during conjunction but not feature tasks
Muggleton et al. (2003)	Feature: color Conjunction: color-orientation	R-FEF L-FEF	Vertex V5	10 Hz for 500 ms	65% MSI	5	FEF is involved in visual selection, in the absence of saccade
Ellison et al. (2004)	Feature: orientation Conjunction: color-orientation	R-PPC R-STG	SHAM on R-PPC or R-STG	R-PPC: 10 Hz for 500 ms R-STG: 4 Hz for 500 ms	65% MSI	5	R-PPC involved in conjunction and R-STG in feature
O’Shea et al. (2004)	Conjunction: color-orientation	R-FEF	Vertex V5	Double-pulse (40 ms interval) at 5 possible delays (from 0 to 120 ms), 40 ms before mask onset	FEF & Vertex: 65% MSI V5: 110% phosphene threshold	12	FEF is involved in visual discrimination, in the absence of saccade
Fuggetta et al. (2006)	Conjunction: color-orientation	R-PPC	Vertex	Single-pulse 100 ms after stimulus onset	85% MSI on average	7	R-PPC involved in conjunction task
Ellison et al. (2007)	Conjunction: color-orientation and motion-orientation	R-PPC R-V5	SHAM on R-PPC or R-V5	10 Hz for 500 ms	65% MSI	7	R-PPC involved in color-orientation R-V5 in motion-orientation
Kalla et al. (2008)	Conjunction: color-orientation	R-FEF R-PPC	No-TMS	Double-pulse (40 ms interval) at 5 possible delays (from 0 to 200 ms)	60% MSI	9	R-FEF involved earlier than R-PPC in conjunction tasks
Muggleton et al. (2008)	Feature: color Conjunction: color-orientation	R-AG L-AG	Vertex No-TMS	10 Hz for 500 ms	65% MSI	8	R-AG but not L-AG involved in conjunction but not feature tasks
Schindler et al. (2008)	Feature: orientation Conjunction: color-orientation	R-PPC R-STG	SHAM on R-PPC or R-STG	R-PPC: 10 Hz for 500 ms R-STG: 4 Hz for 500 ms	65% MSI	5	R-STG processes the left part of the search array presented contralateral R-PPC involved for left visual field when full-fields array
Kalla et al. (2009)	Feature: color Conjunction: color-orientation	DLPFC	Vertex V5	TBS: 50 Hz bursts at 5 Hz	40% MSI	12	DLPCF involved in conjunction but not feature tasks
Dugué et al. (2011)	Conjunction: L vs T and L vs +	V1/V2	Stimulus at non-retinotopic location	Double-pulse (25 ms interval) at 8 possible delays (from 100 to 450 ms)	55% MSI	11	V1 involved at late delays (feedback) during conjunction but not feature tasks
Lane et al. (2011)	Feature: shape Conjunction: color-orientation	R-PPC	SHAM on R-PPC	10 Hz for 500 ms	65% MSI	12	R-PPC involved in conjunction and feature tasks when participants have to point to the target, but only conjunction when button press response
Muggleton et al. (2011)	Conjunction: color-orientation	R-FEF L-PPC	No-TMS	10 Hz for 500 ms	60% MSI	8	L-PPC involved when manual motor response required
Dugué et al. (2015a)	Conjunction: L vs T and L vs +	V1/V2	Stimulus at non-retinotopic location	Double-pulse: one at 312.5 ms and one 13 possible delays (from 112.5 to 437.5 ms)	55% MSI	10	V1 involved periodically during conjunction but not feature tasks (∼6 Hz)
Yan et al. (2016)	Feature: color Conjunction: orientation of triangles	R-DLPFC R-PPC	Vertex on another group of participants	Double-pulse (100 ms interval) 200 ms before search array onset	51% MSI on average	16	DLPFC is involved in the conjunction search, while the PPC is involved in the feature search
Present study	Conjunction: L vs T	R-FEF	Vertex	Double-pulse (25 ms interval) at 9 possible delays (from 50 to 450 ms)	52% MSI on average	21	FEF involved periodically during a conjunction task (∼6 Hz)

For each study we report the behavioral manipulation, the stimulation parameters: tested region (R, Right; L, Left; V5, MT area; PC, parietal cortex; PPC, posterior parietal cortex; AG, angular gyrus, part of the PPC; STG, superior temporal gyrus; DLPFC, dorsolateral prefrontal cortex, right hemisphere), control condition, type of stimulation (TBS, theta burst stimulation; note: otherwise mentioned, the reference is the onset of the visual stimuli) and the intensity of the stimulation (MSI, maximum output stimulation intensity of the TMS machine), the amount of participants for which data were analyzed (*n*), and finally, the authors’ conclusions.

## Materials and Methods

### Participants

Twenty-three participants (7 women), aged 24–38 years old, were recruited. Two did not complete the experiment because of discomfort due to the stimulation. All participants gave written informed consent before the experiment. Standard exclusion criteria for TMS were applied. The study was approved by the local ethics committee Sud-Ouest et Outre-Mer I (protocol 2009-A01087-50) and followed the Code of Ethics of the World Medical Association (Declaration of Helsinki), and international guidelines and safety rules for TMS experiments (Rossi et al., 2009).

### Stimulus procedure

Participants were placed 57 cm from the screen (36.5 × 27° of visual angle) in a dark room. Their head was maintained by a chinrest and headrest. They performed 26 blocks of 72 trials each. One block was used for practice. One block allowed the determination of the stimulus onset asynchrony (SOA) to reach ∼70% correct, using a staircase procedure. Then, 24 blocks corresponded to the main experiment: 4 blocks with no TMS, 10 blocks with TMS applied over the FEF, and 10 blocks applied over the Vertex (control; see TMS procedure).

**Figure 1.**
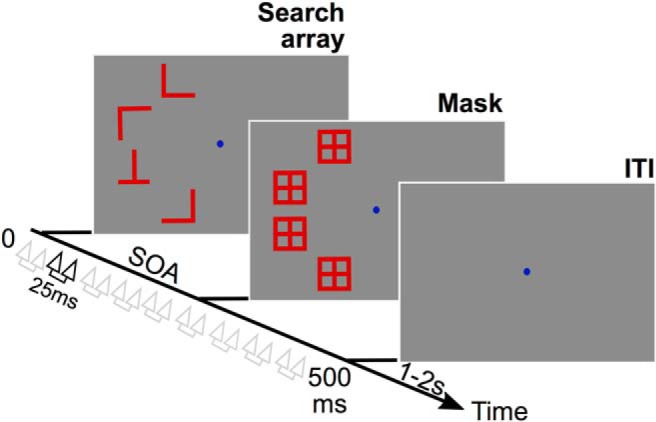
Experimental protocol. While participants performed a visual search (finding a T letter among L’s), they were stimulated over the right-FEF or the Vertex (control) with a double-pulse of TMS (25 ms interval) applied at random delays between 50 and 450 ms (50 ms increments) after the search array onset.

Participants performed a difficult visual search (Fig. 1; same procedure as by Dugué et al., 2011, 2015a): report the presence or absence of a target letter T, among distractor letters Ls (1.5 × 1.5°). On each trial, four stimuli were presented on the left hemifield at constant eccentricity (6°): either four L’s (target absent trials) or three L’s and one T (target present trials), randomly presented in four orientations (0, 90, 180, or 270° from upright). Stimuli were always presented in the left visual field. Behavioral responses were evaluated as per d′, hit rates (correct responses when target present), false-alarm rates (incorrect responses when target absent) and criterion. Participants were asked to respond accurately, and with no time pressure, by pressing a key on the keyboard.

The SOA, i.e., the delay between search array onset and mask onset, was predefined for each observer to achieve ∼70% correct (85 ± 8 ms). The total trial duration (including search array and masks) was 500 ms for all participants. The intertrial interval was randomized between 1 and 2 s, which, adding to the total duration of the trial, limited cumulative effects of stimulation.

### TMS targeting and delivery

TMS pulses were delivered using a 70 mm figure-of-eight coil (biphasic stimulator, Magstim Rapid^2^). A structural T1-weighted MRI scan (3T Philips, flip angle = 8°, TR = 8.1 ms, TE = 3.7 ms, FOV = 240 × 240 mm, voxel size = 1 mm isotropic) was acquired for 11 participants at the imagery platform of the CerCo (Toulouse University). For these participants, the right FEF was localized on each individual MRI using averaged Talairach coordinates *x* = 31, *y* = −2, *z* = 47 (Paus, 1996), and a 0.5 radius spherical region-of-interest (ROI; same procedure as by Chanes et al., 2012, 2013). The final MRI was uploaded into a frameless stereotaxic system and reconstructed in 3D for its use in an online TMS neuronavigation system (eXimia NBS system, Nextim).

Participants were all wearing an EEG cap to help localize the stimulated ROI on the surface. For the 11 participants whose FEF was localized using individual anatomic MRIs, the stimulation site was marked by a small sticker placed on the EEG cap. For the remaining eight participants (for whom we were unable to record a structural MRI), the stimulation ROI was determined as the barycenter of the region of stimulation from the 11 previous participants, and also marked by a small sticker placed on the EEG cap to assist in the TMS coil positioning. The stimulation region for these participants was situated between the electrodes F2 and FC4 (MCN EEG system).

The TMS coil was placed tangentially to the skull and its handle oriented 45° in a rostral-to-caudal and lateral-to-medial orientation. The stimulation intensity started at 50% of the TMS machine maximal output, and was then adjusted just below the threshold of facial and temporal muscle activation (average intensity across participants = 52 ± 2% SEM). For comparison, Dugué et al. (2016) reported an average intensity across participants = 58 ± 2% SEM of TMS machine output intensity (same TMS machine) for the phosphene threshold (perceived 50% of the time).

The Vertex was used as a stimulation control site for nonspecific TMS effects such as clicking noise and tapping sensation. This region was localized for each participant as the region under electrode Cz on the EEG cap (O’Shea et al., 2004). Note that this Vertex versus FEF stimulation conditions were blocked (randomization was not possible because it would have necessitated two TMS machines). Moreover, the Vertex stimulation, unlike the FEF stimulation, was not lateralized to the right hemisphere. Altogether, it is thus clear that the participants could distinguish between the different stimulation conditions. Yet, we ensured participants were unaware of the research question and of the relevance of the stimulation sites.

### TMS procedure

Double-pulses of TMS (25 ms interval) were applied at random delays after search array onset to interfere with search processing (9 possible delays, from 50 to 450 ms, 50 ms increments; Fig. 1). Note that for most of the TMS delays, the search array was no longer on the screen when TMS was applied over the FEF. TMS thus interfered with an internal representation of the array, still being processed by the visual and attentional processing stream. Additionally, the maximum delay between the stimulus offset and the last TMS pulse was kept under 400 ms. This time interval is less than typically associated with a demand for working memory (>600 ms; Phillips, 1974).

Double-pulses were chosen (vs single-pulses) based on the results of Dugué et al. (2011). Double-pulses, although often separated by coarser intervals, are commonly used in the literature to increase the potential effect of the stimulation on performance (Juan and Walsh, 2003; O’Shea et al., 2004; Kalla et al., 2008; Dugué et al., 2011, 2015a; Yan et al., 2016). Right-FEF and Vertex stimulations were blocked. Half of the participants (randomly assigned) performed the right-FEF blocks first, whereas the other half started with the Vertex ones. Participants performed 80 trials per stimulation delay and condition, for a total of 1440 trials per session (2 h), and 720 double-pulses per stimulated site.

### Reanalysis of two previously published datasets

In the current study, we compare the effect of TMS applied at various delays over the FEF during a difficult visual search task, with the results of two previously published studies using the same search task (L vs T), while TMS was applied over the occipital pole (V1/V2).

The first dataset comes from the published study by (Dugué et al., 2011). Based on phosphene mapping, double-pulses of TMS (25 ms interval) were applied at one of various delays (8 possible delays from 100 to 450 ms, 50 ms increments) to a consistent brain location in retinotopic areas (V1/V2). The search array was presented either at the location affected by the TMS pulses (phosphene region) or in the symmetric region in the opposite hemifield (retinotopically-defined control region). Thus, the stimulation was identical over the cortex but was either interfering with the stimulus, retinotopic location (phosphene condition), or not (control condition; for further methodological details, see Dugué et al., 2011).

The second dataset comes from the published study by Dugué et al. (2015a). In this study, the authors followed the same procedure as by Dugué et al. (2011). The only difference is the way the two pulses were administered. In each trial, one pulse remained fixed at a latency of 312.5 ms after the search array onset, based on the main effect found previously by Dugué et al. (2011). The second pulse was applied at 13 other possible delays before or after the first pulse (112.5, 137.5, 162.5, 187.5, 212.5, 237.5, 262.5, 287.5, 337.5, 362.5, 387.5, 412.5, or 437.5 ms after stimulus onset; Dugué et al., 2015a). Note that, despite some methodological differences between the two V1 datasets, their reanalysis is an important test of replicability.

### Fourier analysis

For all three studies, we first calculated *d′*, criterion, hit rates and false-alarm rates (specifically, the normal inverse distribution of hit and false-alarm rates, i.e., correctly and incorrectly, respectively, reporting the presence of the target) as main dependent variables. For each of these four measures, we calculated a modulation index by subtracting the main stimulation condition and the control condition. In the current study, the modulation index was the difference between the right-FEF and the Vertex condition trials. In the two previously published V1 studies, the modulation index was the difference between the phosphene condition and the control condition trials (see previous section). In other words, in all three cases, negative values corresponded to a target region-specific impairment of performance by TMS.

**Figure 2.**
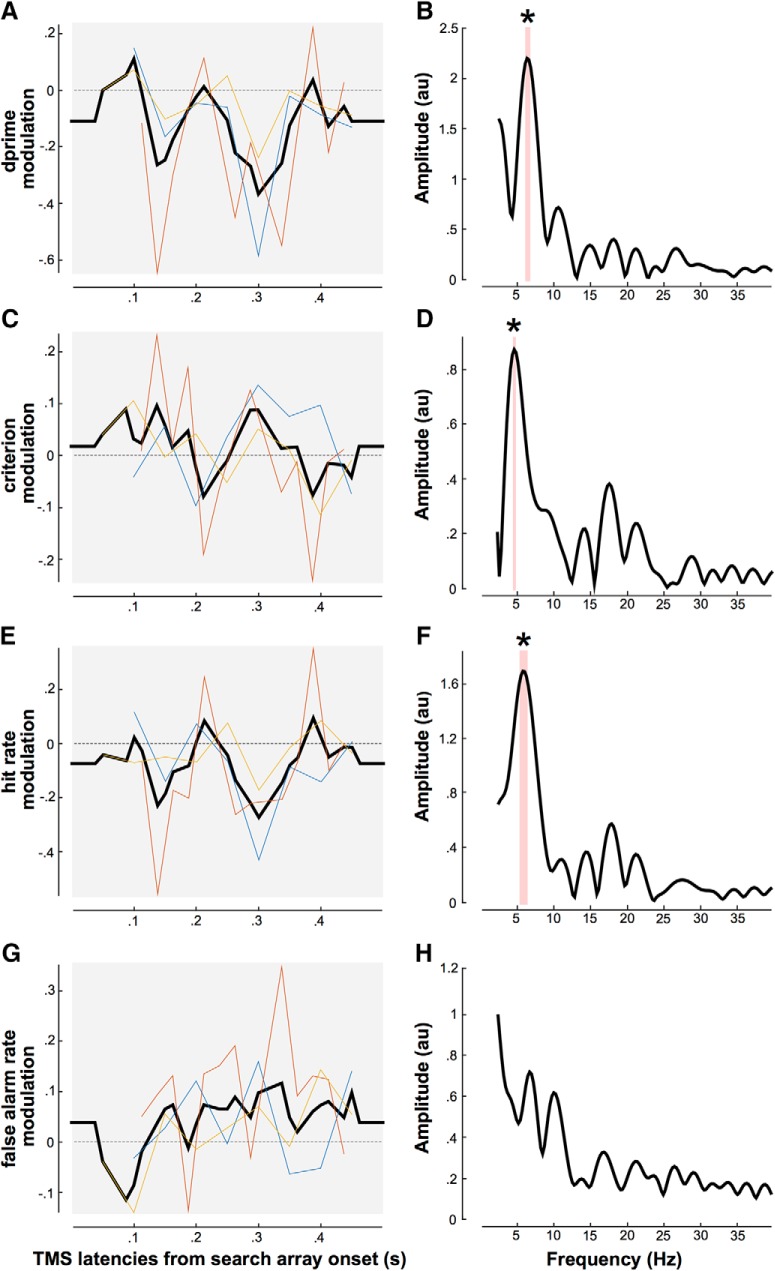
TMS modulates attentional search periodically. ***A***, *D′* modulations (test–control condition) are represented as a function of TMS latencies from the search array onset. The color lines represent each individual study [yellow, current FEF study; blue, first V1 study (Dugué et al., 2011); red, second V1 study (Dugué et al., 2015a)]. The black line is the average across all three studies. ***B***, Amplitude spectrum obtained by FFT decomposition of the averaged data across the three studies. The red shaded area represents the significant spectral components and the *peak at 6.3 Hz (*p* < 0.05). ***C***, ***E***, ***G***, represent criterion, hit rates, and false-alarm rates modulations, respectively (same representation as in ***A***). ***D***, ***F***, ***H***, represent their corresponding amplitude spectra. The red shaded area represents the significant spectral components and the *peaks at 6 Hz and 18 Hz for criterion and 5.8 Hz for hit rates (*p* < 0.05).

We first combined the results from all three studies to investigate the overall TMS modulation of attentional performance during the difficult search task. Because the TMS pulses were not applied at the same delays across the three studies, we first oversampled each individual modulation time course every 12.5 ms using a linear interpolation. We then averaged all three datasets together (Fig. 2*A*,*C*,*E*,*G*). On this pooled dataset, we performed a fast Fourier transform (FFT; Fig. 2*B*,*D*,*F*,*H*) on padded data (using the average value) to get a 4000 ms segment. Note that we also did the analysis on non-padded data and obtained comparable results. Bootstrapping assessed the significance of each frequency component: the simulations were obtained by shuffling the labels of TMS delays, following the null hypothesis that the modulation index was independent of TMS latency (100,000 iterations). For each of the 100,000 surrogate amplitude spectra, we selected the maximum value across frequencies (thus addressing the problem of multiple comparisons). The surrogates were then ranked in ascending order. The 95,001th value was considered as the limit of the 95% confidence interval (CI; *p* < 0.05).

**Figure 3.**
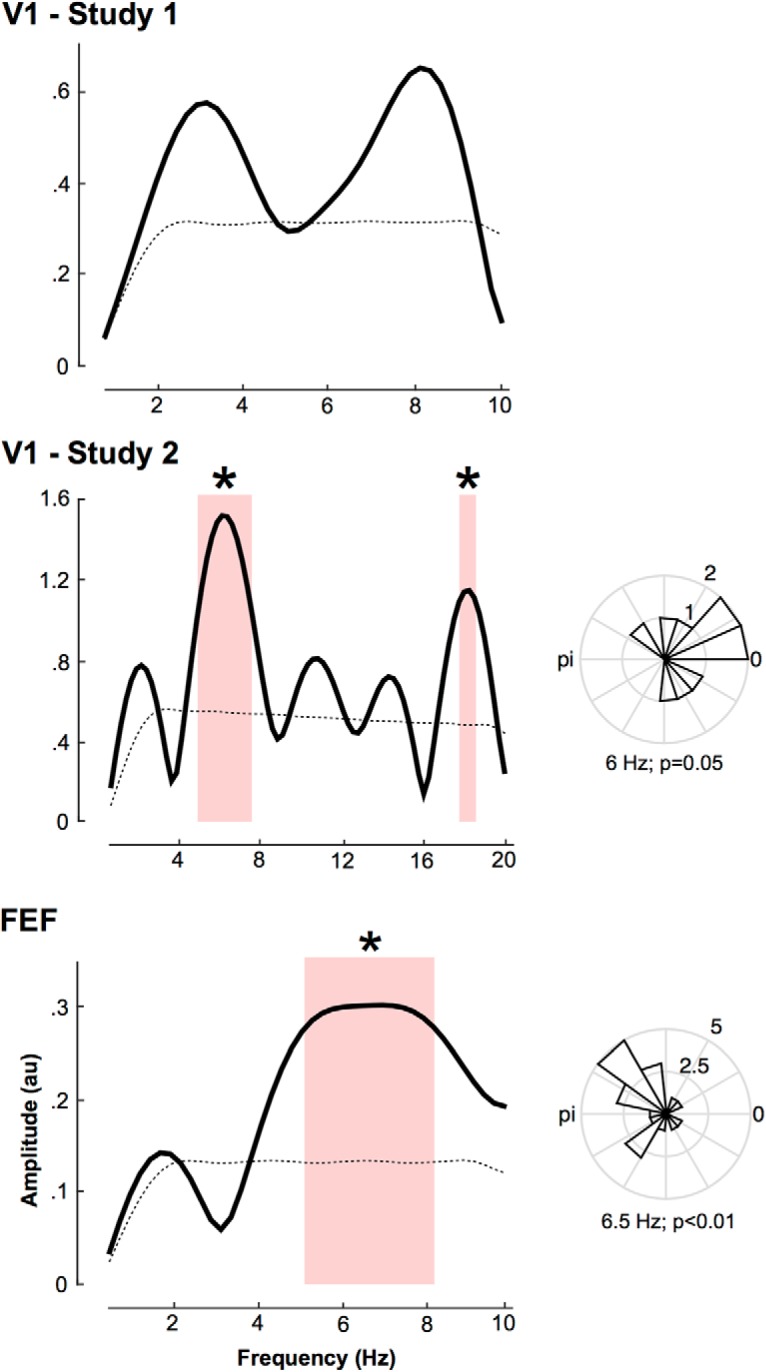
Attentional periodicity in V1 and FEF. For each study, the graphs in the left column represent the amplitude spectra obtained by FFT decomposition of the averaged performance (as per hit rates modulation; see Materials and Methods) across participants. Note the distinct frequency axis in the middle, because of the increased time resolution (and corresponding Nyquist frequency) in that study. The bottom, dashed black line represents the amplitude spectrum of the surrogate distribution. The red shaded area represents the significant spectral components and the *peaks at 6 Hz and 18 Hz for the second V1 study and 6.5 Hz for the FEF study (*p* < 0.05). The right column represents the phase distribution of the peak frequency across participants. *P* values are obtained from Rayleigh test for non-uniform distribution of circular data.

We then looked at the amplitude spectra of each single study using FFT decomposition of the hit rates modulation index only (the previous results indicating that hit rate was the most relevant of the four measures). Note that the method used here to investigate the temporal dynamics of FEF attentional processing during visual search is similar to the one used in the first V1 study (Dugué et al., 2011). To allow for a fair comparison between the two studies, it is critical to perform the same spectral analysis on both datasets, which had not been done at all by Dugué et al. (2011). On the three individual datasets, we performed an FFT on padded data (using the average value) to get a 4000 ms segment. Note that we also did the analysis on non-padded data and obtained comparable results. Here, the significance of each oscillatory component was assessed by nonparametric statistics. Monte Carlo simulations were performed under the null hypothesis that the hit rates modulation was independent of TMS latency (100,000 iterations). For each iteration, we recomputed the grand-averaged curve of the difference of hit rates between test and control conditions, and its amplitude spectrum. For each surrogate, we selected the maximum value across the significant frequencies obtained in the combined analysis (Fig. 2*F*; i.e., 5.5–6.3 Hz). We then sorted these surrogates in ascending order and calculated confidence intervals and the corresponding *p* values (Fig. 3, left column).

Finally, for each participant in the FEF study as well as the second V1 study (Dugué et al., 2015a; we did not perform the analysis for the first V1 study, Dugué et al., 2011, because we did not find a significant peak in frequency in the previous analysis), we looked at the phase distribution across participants for the significant frequency peak observed in the previous amplitude spectra (Fig. 3, right column). We computed Rayleigh statistical test to evaluate the non-uniformity of the phase across participants.

## Results

To test the prediction that difficult visual search periodically involves low- and high-level regions along with iterative attentional selection, we conducted a TMS experiment in which we interfere with the FEF at various delays while observers performed a task in which they have to report the presence or absence of the letter T among letter L’s. The modulation of *d′*, criterion, hit rates, and false-alarm rates was calculated as the difference between the right-FEF and the Vertex (control) stimulation. These results were combined with the results of two previously published studies (Dugué et al., 2011, 2015a) using the same search task (L vs T), while TMS was applied either over the retinotopic location of the early visual cortex corresponding to the search array location, or over the symmetric (control) location (see Materials and Methods). Note that a comprehensive review of the literature on TMS studies of attention during visual search reveals that no other study had the necessary temporal sampling resolution for such an investigation (i.e., single- or double-pulses of TMS sampling a large time window at multiple delays on separate trials; Table 1). Figure 2*A* represents the combined *d′* modulation across all three studies, as a function of the delays at which TMS was applied during the difficult search task. Similarly, Figure 2*C* represents the modulation of the combined criterion, Figure 2*E* the hit rates, and Figure 2*G* the false-alarm rates.

We further investigated the temporal dynamics of these performance modulations. An FFT applied to the combined dataset revealed a significant peak at 6.3 Hz for the *d′* modulation (Fig. 2*B*). A peak at 4.8 Hz was similarly observed for the criterion modulation (Fig. 2*D*). To understand further the observed periodicity in both *d′* and criterion, we performed the same analysis on the modulation of hit and false-alarm rates. Interestingly, we observed a significant peak frequency at 5.8 Hz for hit rates (Fig. 2*F*), but no peak in frequency for false-alarm rates (Fig. 2*H*). Together, these results suggest that the observed periodicity in both *d′* and criterion is because of the periodic modulation of hit rates around the same frequency. This is because *d′* = *z*(hit) − *z*(fa), and criterion = [*z*(hit) + *z*(fa)]/2, where *z* is the inverse normal cumulative distribution function. If sensitivity (*d′*) was the key variable that is periodically modulated by theta oscillations (i.e., in the absence of a criterion fluctuation), then both hits and false alarms should oscillate (with an opposite phase). Similarly, if sensory threshold (criterion) was the key variable (in the absence of *d′* modulations), then hits and false alarms should also both oscillate (in phase). The most likely explanation, therefore, is that oscillations directly affect hit rates, which is then indirectly (because of the two equations above) reflected in *d′* and criterion modulations. In sum, these results suggest that TMS periodically interferes with search performance as per hit rates at the theta frequency. In the next analysis, we thus focus on the modulation of hit rates only.

Critically, to understand the origin of this overall, descriptive effect, we performed the same frequency decomposition on each individual dataset (Fig. 3). In the first V1 study (Dugué et al., 2011), we did not observe a significant peak in frequency (although the shape of the amplitude spectrum, as well as the time course of hit rates shown in Fig. 2*E*, are globally comparable with the other 2 studies). However, for both the second V1 study (Dugué et al., 2015a) and the current FEF study, we observed significant frequency peaks: 4.5–7.3 and 17.6–18.3 Hz (we speculate that this second peak is because of aliasing, more susceptible at higher frequencies) for the second V1 study, and 5–8.3 Hz for the FEF study. This is all the more striking knowing that their respective methodologies had some differences. These methodological discrepancies are likely to explain the small frequency differences we observed between the studies (see Materials and Methods). Importantly, the theta-frequency spectral peak was present across observers, as measured by phase alignment, i.e., there was a significant concentration of the phase across observers (Fig. 3, right column; second V1 study, Rayleigh test of the 6 Hz component: *z* = 2.9, *p* = 0.05; FEF study, Rayleigh test of the 6.5 Hz component: *z* = 4.6, *p* < 0.01). The observed periodicity is thus not a mere effect of performance averaging across participants.

## Discussion

Using TMS applied at various delays while observers performed a difficult, attentional search task, we showed that both V1 and the FEF are involved periodically during the search, at the theta frequency (∼6 Hz). The FEF periodicity is consistent with two recent findings. Using a cueing procedure, an electrophysiological study in monkey (Fiebelkorn et al., 2018) and an intracranial recording study in humans (Helfrich et al., 2018) both showed rhythmicity of attentional sampling at the theta frequency. Interestingly, similarly to the present study, they linked theta rhythms in frontal cortex to behavioral performance.

The reanalysis of the first and second V1 studies was to address a timely concern in the TMS literature, i.e., replicability (Biel and Friedrich, 2018; Lopez-Alonso et al., 2018). Although no significant peak frequency effect was found in the first TMS study, which may be due to various experimental factors, interestingly, a trend was observed in the theta frequency range. Further studies will be necessary to understand the discrepancy in the strength of the effect between the first and second V1 studies.

Although a direct link between the two periodicities is not demonstrated here, we may speculate these findings indirectly support the idea that visual search tasks are processed by a hierarchical system involving periodic, iterative connections between low- and high-level regions until target recognition. This hypothesis is in line with the large accumulation of evidence that attention acts via feedback to sensory areas (Motter, 1994; Mehta et al., 2000; Schroeder et al., 2001; Kastner and Pinsk, 2004; Bressler et al., 2008; Fiebelkorn et al., 2018; Helfrich et al., 2018). An additional prediction made by such hierarchical two-way processing stream is that if both the low- and high-level regions are periodically sending information to each other, there should also be a phase lag between their respective modulations. Unfortunately, because the results were obtained from independent studies (different participants and sample sizes) and the peak frequency was not the same across the studies, we were not able to compare their phases. In the future, one could perform an experiment in which the same observers are stimulated at various delays over the FEF and V1, while performing the same difficult search task. This would allow the characterization within the same participants of the respective temporal dynamics of V1 and FEF, and their interaction, during difficult visual search.

Oscillations in behavioral performance have been the topic of a large, recent body of research. Two rhythm frequencies have been reported (VanRullen, 2016), i.e., alpha (∼10 Hz) and theta (∼7 Hz). It has been suggested that while the alpha rhythm reflects an intrinsic, sensory rhythm, sampling information at a single location, theta rather reflects attentional exploration, sampling information at multiple locations (Dugué and VanRullen, 2017). This hypothesis is in line with a recent TMS experiment in humans in which attentional exploration was explicitly manipulated using a cueing paradigm (Dugué et al., 2016). By applying TMS at various delays over V1, the authors demonstrated that performance in a 2-AFC orientation discrimination task was modulated by TMS periodically at the theta frequency (∼5 Hz) only when attention had to be reallocated from a distractor to a target location. Interestingly, recent electrophysiology studies in the visual cortex of macaque monkeys showed attention-related theta rhythms in visual cortex, including areas V1 and V4 (Kienitz et al., 2018; Spyropoulos et al., 2018).

One might wonder whether the observed periodicity in all the previously described TMS studies (including the present one) is because of a true, intrinsic property of the attention system, which processes information periodically, or whether it is actually induced by the TMS. One critical piece of evidence in favor of the former is that in (Dugué et al., 2015a) the authors not only observed a periodicity in behavioral performance due to the stimulation, but also showed in independent trials without TMS (but in the same participants) that brain oscillations (as measured by EEG) at the same frequency (∼6 Hz; theta) correlated with search performance. Consequently, oscillations likely reflect a periodicity in cortical excitability, and TMS is thus able to probe the system at different excitability states. More generally, interventional methodologies, such as TMS, have the critical advantage to go beyond mere correlational evidence and inform neural processes to establish causal links between cortical excitability and behavioral processing.

In the present study, we show that the FEF is involved at the theta frequency during this attentional search. Previous studies investigating the spontaneous activity of the frontoparietal region (Rosanova et al., 2009), and the role of the FEF in attentional search in monkeys (Buschman and Miller, 2007, 2009) and humans (Phillips and Takeda, 2010; Chanes et al., 2013; Quentin et al., 2015), however, showed periodicity in the low (13–24 Hz) and high (36–56 Hz) beta frequency range. Given the use of multiple delays in the different TMS studies presented here, frequencies >10 Hz could not be characterized. Thus, we cannot rule out that other, higher frequencies are related to attentional sampling during this difficult visual search. It is even possible that the use of 25 ms TMS double-pulses in two of the three studies (Dugué et al., 2011; and current) could have assisted in recruiting some of these oscillations (∼40 Hz); the fact that compatible results were obtained in the third study (Dugué et al., 2015a) without this 40 Hz periodicity, however, indicates that it could be instrumental but not strictly necessary for recording TMS modulations of visual search.

In addition to the theta (VanRullen et al., 2007; Busch and VanRullen, 2010; Landau and Fries, 2012; Fiebelkorn et al., 2013, 2018; VanRullen, 2013; Song et al., 2014; Huang et al., 2015; Landau et al., 2015; Dugué et al., 2015a,b, 2016, 2017; Helfrich et al., 2018) and beta (as discussed in the previous paragraph) oscillations, there is a great amount of evidence for a modulation of alpha oscillations (synchronization/desynchronization) because of spatial allocation of attention (Worden et al., 2000; Sauseng et al., 2005; Thut et al., 2006; Rihs et al., 2007; Jensen and Mazaheri, 2010; Samaha et al., 2016; Brüers and VanRullen, 2018). It is thus plausible that more than one oscillation coexists and interacts during visual attention, and visual search in particular. Studying the interplay between theta, alpha, and beta oscillations in attentional deployment is an exciting question for future research.

In the present study, we investigated the temporal dynamics of V1 and FEF during attentional search, and revealed that both regions are involved periodically at the theta frequency. This study brings convincing, converging evidence, together with multiple studies using various approach including psychophysics, EEG and TMS, and analysis tools, in favor of a theta, intrinsic rhythm as the support of attentional exploration.

## Synthesis

Reviewing Editor: Bradley Postle, University of Wisconsin Decisions are customarily a result of the Reviewing Editor and the peer reviewers coming together and discussing their recommendations until a consensus is reached. When revisions are invited, a fact-based synthesis statement explaining their decision and outlining what is needed to prepare a revision will be listed below.

Reviewer 1:

The present manuscript, titled “Contribution of FEF to attentional periodicity during visual search: a TMS study,” describes a periodic disruption in behavioral performance following TMS applied over FEF, a higher-order cortical region involved in directing attentional selection and eye movements. TMS was applied at various delays during a search task (relative to onset of the search array). The results indicate that theta rhythms in FEF are causally linked to visual search (i.e., looking for a target embedded in distractors). These findings are then discussed in the context of earlier results, which showed a similar pattern of theta-rhythmic interference when TMS was applied over V1. A limitation of the study, acknowledged by the authors, is that they cannot test whether there is a functional relationship between the theta rhythms in higher-order and sensory regions. Even without that piece, the study makes a meaningful contribution to the existing literature. While a couple of recent papers have linked theta rhythms in frontal cortex to attentional sampling, those studies did not include causal manipulations. This study therefore expands on previous findings, showing that theta rhythms in FEF are causally involved in mediating attentional sampling, specifically during visual search. There are only a couple of issues that I would like the authors to address.

(1) Given the setup of the behavioral task, I'm having some difficulty with interpreting the results. The authors suggest that their findings are consistent with FEF being periodically involved during the search task, but what in particular is being interrupted by TMS? TMS was typically applied when the search array was no longer on the screen, so how can it be characterized as disrupting ‘visual search’? Was the TMS interrupting visual search of a memory trace?

(2) Although two recent studies in Neuron (Fiebelkorn et al., 2018 and Helfrich et al. 2018) are referenced as evidence that attention samples periodically, there is no mention that these studies have similarly linked theta rhythms in frontal cortex to behavioral performance. That seems highly relevant. The authors might also want to consider discussing their results relative to neurophysiological evidence of attention-related theta rhythms in sensory cortex (e.g., Spyropoulos et al., PNAS, 2018; Kienitz et al., Current Biology, 2018).

Reviewer 2:

In this manuscript, authors employ a causal approach (dual pulse TMS to interfere/modulate visual search) to study periodical attentional sampling operating on the human right FEF. The study provides solid evidence of iterative (rhythmic) theta attentional sampling demonstrated by periodic increases (and periodic decreases) of visual search performance at a 7Hz frequency on the right FEF (controlled by a TMS active condition delivered to the vertex). In the second part of the manuscript, authors re-enact data from two prior TMS studies on V1 (published in 2011 and 2015a) using a very similar task and somehow similar TMS approach in different populations of participants. They report similar periodical sampling phenomena, leading authors to quite explicitly suggesting iterative top-down interactions between FEF and V1 theta rhythms during visual search tasks. The manuscript is very well written, and contains rather convincing evidence of theta right FEF periodicity during visual search.

Major point

My only major point is that on the basis of independent datasets obtained in separate studies performed on different populations (with methodological differences, which in some cases could be deemed substantial), I find somehow misleading (or at best highly risky) to suggest iterative top down interactions between V1 and FEF theta rhythms, As authors themselves indicate in the discussion, a full demonstration of such a relation (ideally causal) between FEF and V1 theta rhythmicity would require an ad hoc performed on the same population of participants which using either EEG alone, coupled TMS-EEG approaches could really demonstrate which area iteratively leads (likely the FEF) and which one follows, and eventually to be able to estimate a phase difference between the two theta rhythms (FEF and V1) compatible with conduction time between these two sites. In absence if such evidence, any suggestion is purely speculative and supported by a theoretical framework rather than demonstrated by actual data.

To satisfactorily address this major point and avoid reader's confusion, I would suggest the authors to limit the current report to their original FEF theta periodicity and eliminate from the manuscript the re-analyses of V1 behavioral outcomes from prior related papers (already made clear in such publications anyway). Any ambiguity with regards to suggesting the possibility that the current manuscript addresses the issue of how FEF and V1 theta periodicity might interact (rather than simply coexist or co-occur in different cohorts of tested healthy participants performing similar but not 100% identical tasks 100% identical TMS interference procedures) should be removed from the text. Authors should verify the Significance statement (see page 1, lines 25-27), the abstract (see page 1, lines 7-8 and line 16-17), the introduction (see page 2, lines 51-54 and lines 61-62) and the results (see page 7, lines 183-186). I don't see any problem to leave the authors speculate in the paper discussion on a probably causally association (and eventually one iteratively driven by the other) between FEF and V1 theta rhythms, be, and if necessary to have them spell out in further detail the type of experiment needed to demonstrate their interactive iterative role during visual search.

Minor points

1. Page1, Line 25 (section on significance statement). The sentence should read “This finding supports the idea ...” instead of “This finding support the idea...”

2. Page 4, Line 85. It is unclear how exactly was the d' estimated from the visual search task (what was taken as hits, misses, false alarms and correct rejections) neither if the authors tested potential modulation of response criterion (beta). Please clarify in the manuscript.

3. Page 4, Lines 88-89. Authors declare an inter-trial time of 500 ms which is somehow short to avoid individual pulses or doublets to build up a frequency on FEF able to modulate excitability. Is there any behavioral, TMS or EEG evidence of facilitation of function due to cumulating pulses during the experiments? Were international guidelines and safety rules of number of pulses and intertrain intervals respected?

4. Page 4, Lines 105-107. Not sure this reviewer understands why participants were wearing an EEG cap to localize the stimulated ROI on the scalp surface if an MRI-based neuronavigation system could be used prior and also online during the experiment. It is also unclear and difficult to understand how was the ROI determined for the eight participants without an MRI

5. Page 5, Lines 109-113. Comparison of TMS intensities provided between this and other studies is unclear as different number of pulses were used. The only transversal reference that could be used to provide a useful comparative estimate of the intensity employed in our the study would be the average of individual intensities relative to individual phosphene or motor thresholds (with single or double pulses). Do authors have this type of information?

6. Page 5, Lines 114-116. The active sham condition delivered on the subject's vertex is very appreciated but it might provide sensory accompanying sensations which are not lateralized (as right FEF pulses are), hence easily perceived as different for participants. Could you briefly comment on the potential impact of this observation?

7. Page 5, Line 119. It is unclear why authors employed 25 ms interval doublets instead of single pulses to probe behavioral periodicity. This strategy might generate episodic short lasting two- cycle high frequency rhythms (with a 25 ms period, hence 40 Hz) within a slower rhythm (theta report in the paper). Please clarify in the manuscript and state the reason for that decision.

8. Page 5, Line 119. To what extent the choice of TMS doublet intervals might more easily reveal theta rather than other (particularly faster) periodic sampling rhythms? In other words, could have this exact TMS chronometric design revealed an implication of high beta FEF rhythms in visual search (as suggested by Buschman and Miller 2007 and 2009)? Please comment in the manuscript.

9. Page 5, Line 122-123. Please can you provide in the text an estimate of the approximate total duration of each experimental session and the total number of delivered pulses (or doublets)?

10. Page 6, Lines 137-147. The TMS design and pulse temporal distribution used in the 2015a V1 paper cited here (with a 1st pulse at a fixed timing and 2nd pulse at changing intervals) seems difficult to compare with the use of 25 ms interval double pulses at different onset times. How can the outcomes of these studies be considered comparable?

11. Page 6, Lines 146-150 and 153-155. As clearly stated here, there were obvious differences across the TMS strategies (egg. control conditions used to calculate d' modulations of TMS sampling intervals and time range) employed for V1 and FEF studies that authors attempt to compare. In light of such differences and being datasets obtained in different populations of participants, is this comparison really fair?

12. Page 7, Lines 191-194. Is really necessary to add Table 1 in order to support the statement that to date no other TMS study had the necessary temporal sampling resolution for an investigation of periodical rhythms in visual search? To this reviewer this fair point can be made with a simple statement or by referencing a review paper. Besides I would not call the findings presented in Table 1 a ‘meta-analysis’ but eventually, a comprehensive review of the literature.

13. Page 9, Lines 211-2016 and Page 11 239-241. Could authors provide an interpretation of frequency differences within fluctuations frequency for the FEF (7 Hz), and the 1st and 2nd TMS V1 studies (5.7 Hz and 7.5 Hz)? Can those differences be simply explained by discrepancies in TMS designs and the use of different control conditions, or they do reflect idiosyncratic physiological contributions?

14. Page 11, Lines 256-264. With regards to this same point, authors should do a better job emphasizing the pros and cons of using TMS pulses delivered at regular intervals to probe either excitability or behavioral modulations, rather than relying on EEG recordings combined with behavioral performance.

15. Page 11, Lines 265-272 and 275-277. In the discussion authors should try harder to reconcile their current findings (7 Hz periodic sampling), with correlational and causal evidence supporting a relation of high beta (25-35Hz) in monkeys and also humans with a top down orienting of attention during visual search. Similarly, the current findings and those invoked from the two 2011 and 2015a TMS-V1 publications should be discussed in relation to robust evidence of posterior alpha synchronization/desynchronization during the allocation of attention in space. Could FEF/V1 theta rhythms, V1/posterior parietal alpha rhythms and FEF high-beta rhythms coexist and serve different attentional modes or subprocesses? Which ones?
